# The Impacts of a Primary Care-Based Dietitian Health Coaching Intervention on Patients’ Health: An Observational Study

**DOI:** 10.1177/15598276261460830

**Published:** 2026-07-05

**Authors:** Amy B. Locke, Brittany L. Sisco-Taylor, Xuechen Wang, Robin L. Marcus, Rebecca K. Delaney, Molly B. Conroy, Maribel Cedillo, Joshua Christensen, Angela Fagerlin, Paul A. Estabrooks

**Affiliations:** 1 Department of Family & Preventive Medicine, University of Utah, Salt Lake City, Utah, USA(AL); 2 Utah Osher Center for Integrative Health, University of Utah, Salt Lake City, Utah, USA(AL, PE); 3 Department of Population Health Sciences, University of Utah, Salt Lake City, Utah, USA(ST, XW, RD, MC, JC, AF); 4 Department of Physical Therapy and Athletic Training, University of Utah, Salt Lake City, Utah, USA(RM); 5 Department of Internal Medicine, University of Utah, Salt Lake City, UT, USA(MC, MC); 6 Department of Health & Kinesiology, University of Utah, Salt Lake City, UT, USA(PE)

**Keywords:** primary care, health coaching, health behavior change, lifestyle, integrative medicine, intervention, quality of health care

## Abstract

The objective of this study was to examine the cardiometabolic health of patients receiving a primary care-embedded dietitian administered health coaching intervention. We performed an observational analysis of clinical and questionnaire data from electronic health records (EHRs) among patients receiving the health coaching, including 3- and 6-month changes health and associations of patient characteristics and intervention exposure with outcomes. Questionnaires included health behaviors at baseline, 3, and 6 months. Biophysical data (weight [kg], BMI, HbA1c, blood pressure), number of coaching encounters, and demographics were also collected. Paired *t* tests and ANOVAs compared changes over time and subgroup differences. The analysis sample included 364 adults. Participants had significant weight reductions (mean decreases of −.96 and −1.07 kg after 3 and 6 months: *p*s < .0001) and BMI (6 months: −0.35 kg/m^2^, *P* = .004). Among patients with diabetes or prediabetes, HbA1c values also significantly decreased (6 months: −0.55%; *P* = .018). Finally, the more coaching visits patients had, the greater their weight loss. Including dietitian-trained health coaches on primary care teams may improve clinical outcomes among patients at risk for or with chronic disease.

“Patients showed significant weight loss 3 months after their initial health coaching encounter and sustained after 6 months.”

## Introduction

Despite continuous advances in medical treatment and therapeutics, poor health and lifestyle behaviors continue to contribute to most of the chronic disease burden and mortality worldwide.^1^ Conversely, healthy lifestyle behaviors, such as a healthful diet, physical activity, and sleep, can prevent many of the most severe chronic diseases (e.g., diabetes, cardiovascular disease).^2^ These behaviors can also improve the well-being and quality of life among individuals already living with chronic conditions. Although patients’ lifestyle behaviors occur and are influenced by many factors outside of the health care system, primary care is an impactful setting along the continuum of patient care where conversations surrounding chronic disease prevention and management often occur.^3,4^

Nationwide shortages among primary care physicians and resulting burnout have led to several enhanced models that incorporate extended team members in this setting. In particular, health coaching is 1 promising intervention that has been applied to many contexts, but it has been poorly defined and yields mixed findings, in part due to the diverse roles and training backgrounds of coaches and the diverse settings in which it has been used.^4^ A recently published Health and Wellness Coaching framework for health professionals aimed to clarify these differences by providing a more concise definition of health coaching.^4^ Specifically, they defined health coaching as a process that focuses on the improvement of physical health by using strategies that support patient identification of health priorities and implementation of self-regulatory tools (e.g., goal-setting, self-monitoring).^4^ The authors also identified that health coaches may be from various professional backgrounds (e.g., nurses, exercise trainers, dietitians).^4^

While there has been substantial focus on health behavior change strategies integrated into primary care,^5,6,7,8,9,10,11,12^ there have been few evaluations of health coaching effectiveness when implemented by health professionals in a primary care setting as part of standard practice. In this paper, we present the findings from an observational cohort evaluation of a registered dietitian-delivered health coaching intervention embedded within primary care. We examined pre-post changes in patients’ weight and cardiometabolic outcomes, and associations with patient characteristics and intervention exposure. We hypothesized that patients receiving health coaching would have improved cardiometabolic health outcomes. We also hypothesized that patients who completed 4 or more coaching visits would experience the greatest improvements. We also examined secondary cardiometabolic outcomes of Body Mass Index (BMI), HbA1c, and blood pressure.

## Methods

### Overview

We examined the clinical outcomes of patients receiving primary care health coaching from a registered dietitian prior to engaging in a health coaching visit (i.e., baseline) and at 3, and 6 months post the initial health coaching visit. We also examined associations of patient characteristics (e.g., demographics and self-efficacy for making lifestyle changes) and intervention exposure (i.e., number of completed coaching visits) with weight loss. Finally, we compared the characteristics of patients that engaged with health coaching and had available data for 3 and 6 month follow-up (n = 364) to the total sample of patients that received health coaching over this period (n = 847; Table 1). The University of Utah Health Sciences Institutional Review Board determined this protocol was exempt. This study also adhered to the Strengthening the Reporting of Observational Studies in Epidemiology (STROBE) guidelines.

**Table 1.** Baseline Participant Characteristics.

Patient Characteristics	Baseline sample (*N* = 847)	Analysis sample (*N* = 364)	*P* Value
Sociodemographic			
Age, y - mean (SD)	49.96 (16.32)	52.59 (15.94)	<0.001
- Median (IQR)	51.00 (36.00 – 63.00)	54.00 (40.00 – 65.25)	​
- Range	18.00 – 92.00	18.00 – 92.00	​
Gender- n (%)	​	​	0.074
Female	552 (65.17%)	250 (68.68%)	​
Male	295 (34.83%)	114 (31.32%)	​
Other	0 (0%)	0 (0%)	​
Race**-** n (%)	​	​	0.108
White/Caucasian	686 (80.99%)	299 (82.14%)	​
Black/African American	21 (2.48%)	11 (3.02%)	​
Asian	29 (3.42%)	16 (4.40%)	​
Native Alaskan/American Indian	3 (0.35%)	1 (0.27%)	​
Native Hawaiian/Pacific Islander	6 (0.71%)	0 (0%)	​
Other	87 (10.27%)	33 (9.07%)	​
Choose not to disclose	13 (1.53%)	3 (0.82%)	​
Unknown	1 (0.12%)	0 (0%)	​
N/A	1 (0.12%)	1 (0.27%)	​
Ethnicity**-** n (%)	​	​	0.022
Hispanic/Latino(a)	90 (10.63%)	28 (7.69%)	​
Insurance status**-** n (%)	​	​	0.139
Commercial	365 (43.09%)	131 (35.99%)	​
Medicaid	31 (3.66%)	12 (3.30%)	​
Medicare	112 (13.22%)	54 (14.84%)	​
N/A	339 (40.02%)	167 (45.88%)	​
Self-rated health**-** n (%)	​	​	0.681
Excellent	17 (2.01%)	8 (2.20%)	​
Very good	106 (12.51%)	53 (14.56%)	​
Good	218 (25.74%)	118 (32.42%)	​
Fair	136 (16.06%)	80 (21.98%)	​
Poor	16 (1.89%)	9 (2.47%)	​
N/A	354 (41.79%)	96 (26.37%)	​
Clinical characteristics			
Weight (kg)- mean (SD)	96.34 (24.99)	97.56 (24.96)	0.218
BMI- mean (SD)	33.64 (7.47)	34.35 (7.46)	0.023
BMI clinical categories- n (%)	​	​	0.110
Underweight	8 (0.94%)	3 (0.82%)	​
Normal	87 (10.27%)	32 (8.79%)	​
Overweight	150 (17.71%)	56 (15.38%)	​
Obese	511 (60.33%)	238 (65.38)	​
N/A	91 (10.74%)	35 (9.62%)	​
Systolic BP- mean (SD) mmHg	125.18 (14.40)	124.80 (13.52)	0.534
Diastolic BP- mean (SD) mmHg	79.29 (8.87)	78.72 (9.11)	0.159
HbA1c- mean (SD)	6.14 (1.19)	6.30 (1.36)	0.005
Health conditions- n (%)	​	​	​
Obesity/Overweight^a^	661 (87.43%)	294 (89.36%)	0.196
Diabetes	96 (11.33%)	60 (16.48%)	<0.001
Prediabetes^b^	230 (41.67%)	102 (40.80%)	0.773
Hypertension	86 (10.15%)	56 (15.38%)	<0.001
Current smoker- n (%)	15 (1.77%)	7 (1.92%)	0.977
Number of health coach visits- mean (SD)	2.15 (2.41)	2.81 (3.24)	<0.001
- Median (IQR)	1 (1, 2)	2 (1, 3)	<0.001
- Range	1 – 23	1 – 23	​

BMI = body mass index; HbA_1c_ = hemoglobin A_1c_; BP = blood pressure.

### Setting and Population

This observational cohort analysis study was conducted between December 2017 and July 2020, among a convenience sample of adult patients from 2 Family and General Internal Medicine clinics within a public university health care system. Patients within these clinics were offered health coaching by their primary care physician after a scheduled clinical appointment, or in a message sent through the patient portal. Health coaching participants were included in the effectiveness analysis if they had at least 1 health coaching visit and had available outcome data across baseline and the 3- and 6-month follow-ups. There was no additional standard protocol for health coaching in terms of number of visits and frequency. Decisions were determined by patient preference and individual health goals, without involvement from the study team.

### Health Coaching

Health coaching was initiated in the health care system in December 2017 and was offered free of charge to all patients seen in primary care. The project started in 1 clinic and expanded to include an additional Family and General Internal Medicine clinic over the course of the project. Patients chose to meet with a dietitian-trained health coach in-person, over telephone, or via telehealth. Coaches were available asynchronously through the patient portal between visits. Health coaches included 6 registered dietitians over the course of this evaluation period. Although the coaches were not National Board Certified Health and Wellness Coaches, each completed extensive training through their dietetics coursework and formal Diabetes Prevention Program Coach Training. Monthly coach meetings further developed coaching skills and worked through difficult cases. Coaches focused on helping patients make desired lifestyle changes including nutrition, physical activity, sleep, and other health behaviors. The coaching visits included evidence-based counseling, motivational interviewing, and goal-setting strategies. Health coaches provided support and opportunities to follow-up on patients self-selected goals and used barrier identification and resolution strategies to support future patient behavior change. After the initial visit, coaches followed up with patients to monitor progress and referred patients to other extended care team members (e.g., care managers, behavioral health, clinical pharmacists) as needed or applicable. Of note, health coaches did not provide medical nutrition therapy as a part of these coaching visits. Patients needing specific dietary advice were triaged to traditional dietitian visits.

### Outcomes and Measurements

The primary source of data used for this evaluation was obtained from the health care system electronic health records which included both at-risk patients and patients with chronic diseases. This included demographic information (age, gender), weight, and Body Mass Index (BMI). Additional data for at-risk patient subgroups who presented with diabetes or prediabetes and hypertension, respectively, included blood sugar (HbA1c) and systolic and diastolic blood pressure. In addition, patients completing coaching visits also provided perceptions of self-efficacy prior to the initial health coaching visit and 6 months after that first visit. These data were collected using a survey with 2 items, each rated on a Likert-scale from 1 (*Not at all sure*) to 4 (*Very sure*): “At the present time, how sure are you that you can make and stay with a regular exercise plan?”, and “At the present time, how sure are you that you can make and stay with changes in your diet?”. These questions allowed the coach to tailor questions based on the individual needs of each patient as well as track progress over time.

### Data Analysis

The minimum clinically significant difference for weight was identified to be a reduction of 5%^13^; the minimum clinically significant difference for HbA1c was a change of 0.5%,^14,15,16^ and for systolic and diastolic blood pressure, a 6-month change of 1.32 and 1.44 mmHg, respectively.^17^ To examine the association of patient characteristics and intervention exposure with weight loss over 6 months, independent *t* tests were used to examine mean subgroup differences for dichotomous variables, and ANOVAs were used for categorical variables. To quantify the association of intervention exposure with weight loss, specifically, ANOVAs were applied to test differences in average weight change after 6 months by number of completed coaching encounters (i.e., patients with 1, 2-3, 4-6, and 7 or more encounters). Lastly, a linear mixed-effects regression model was used to examine whether the number of coaching visits (entered as a linear term) predicted 6-month weight change after adjusting for patients’ baseline demographic and clinical characteristics. Covariates were age, gender, and baseline clinical risk factors of a diabetes diagnosis (yes/no), prediabetes HbA1c value (yes/no), and hypertension (yes/no). A model including health coaching visits as a quadratic term was also evaluated; however, the model fit was poor and therefore only results from the linear model are discussed. All statistical analyses were performed using R (version 4.1.0). A significance level of .05 was used for all statistical comparisons.

## Results

A total of 847 people engaged with health coaching over the evaluation period (65% female, 81% non-Hispanic White; 11% Hispanic). Of those, 364 (69% female, 82% non-Hispanic White; 8% Hispanic) had data available prior to the initial health coaching visit and at 3- and 6-month follow-up. Table 1 provides a comparison of baseline characteristics of those with complete data to the broader sample that engaged in health coaching. Statistical comparisons suggested there were fewer participants identifying as Hispanic/Latinx in the analysis sample as compared to the baseline sample. Participants in the analysis sample were at higher risk for chronic disease than those in the baseline sample; on average, study participants presented with higher BMIs and HbA1c values, and they were also more likely to have diagnoses of type 2 diabetes or hypertension.

Of the adult patients who received health coaching, 364 participants (Mean age = 52.6 years; 69% Female; 82% White; 8% Hispanic/Latino) met the inclusion criteria of having at least 1 health coaching visit and had complete data for the primary outcomes at 3 and 6 months were included in this study’s analysis. Of these individuals, most (53%) had a return coaching visit. Most participants (65.4%) had baseline BMIs in the obese range, and 15.4% had overweight. Patients’ primary goal for coaching was weight loss (69% of participants).

Table 2 presents the mean changes in primary and secondary outcomes across the 3 time points. Following the initiation of health coaching, patients showed statistically significant weight reductions after both 3 and 6 months. Reductions in BMI were also statistically significant after 3 and 6 months (*p*s<.001 and .004). Among a subgroup of 53 at-risk patients who had either type 2 diabetes (i.e., diagnoses pulled from the ICD-10) or prediabetes (i.e., HbA1c values of 5.7 to 6.4 range, without a diabetes diagnosis), there was also clinically significant reductions in HbA1c after 6 months of initiating health coaching (*P* = .017), and a statistically significant change at 3 months (*P* = .018). Patients with diabetes saw greater improvements as compared to the patients with prediabetes.

**Table 2.** Changes in Primary and Secondary Health Outcomes for Patients Enrolled in Primary Care-embedded Health Coaching.

Outcome	Time Point	Mean (SD)	Mean change from baseline (95% CI)
Primary outcome			
Weight (kg) (n = 364)	Baseline	97.56 (24.96)	​
	3 months	96.60 (24.90)	−0.96 (−1.34, −0.57)
	6 months	96.49 (24.85)	−1.07 (−1.61, −0.53)
Secondary outcomes			
BMI (n = 288)	Baseline	34.53 (7.55)	​
​	3 months	34.15 (7.52)	−0.38 (−0.55, −0.22)
​	6 months	34.18 (7.53)	−0.35 (−0.59, −0.11)
HbA_1c_ (n = 53)	Baseline	7.64 (1.93)	​
	3 months	7.23 (1.73)	−0.41 (−0.74, −0.07)
	6 months	7.09 (1.50)	−0.55 (−1.00, −0.10)
HbA_1c_^a^ (Diabetes)	Baseline	8.39 (1.93)	​
(n = 36)	3 months	7.78 (1.84)	−0.61 (−1.09, −0.13)
​	6 months	7.54 (1.60)	−0.84 (−1.49, −0.20)
Systolic BP- mmHg^b^ (n = 37)	Baseline	132.10 (13.39)	​
	3 months	135.50 (15.70)	3.41 (−2.51, 9.32)
	6 months	132.90 (14.78)	0.84 (−5.39, 7.06)
Diastolic BP- mmHg^b^ (n = 37)	Baseline	82.41 (8.53)	​
	3 months	81.68 (9.96)	−0.73 (−4.50, 3.04)
	6 months	81.95 (11.14)	−0.46 (−4.74, 3.82)

BMI = body mass index; HbA_1c_ = hemoglobin A_1c_; BP = blood pressure.

^a^Sample included a subgroup of patients with a Type 2 diabetes diagnosis (n = 36) at study baseline.

^b^Sample included a subgroup of patients (n = 37) with diagnosed hypertension at study baseline.

**Figure 1.**
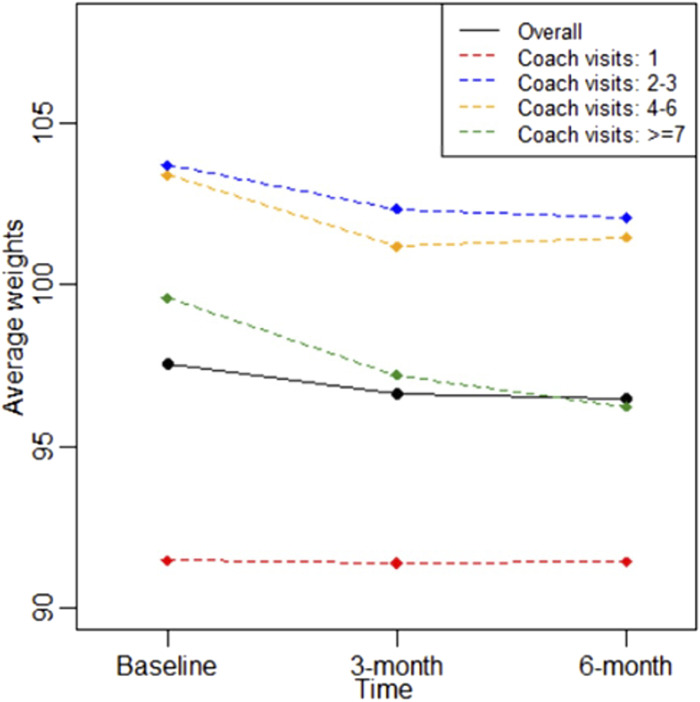
Average weight reductions after 6 months of health coaching by patients' total number of coaching encounters.

**Figure 2.**
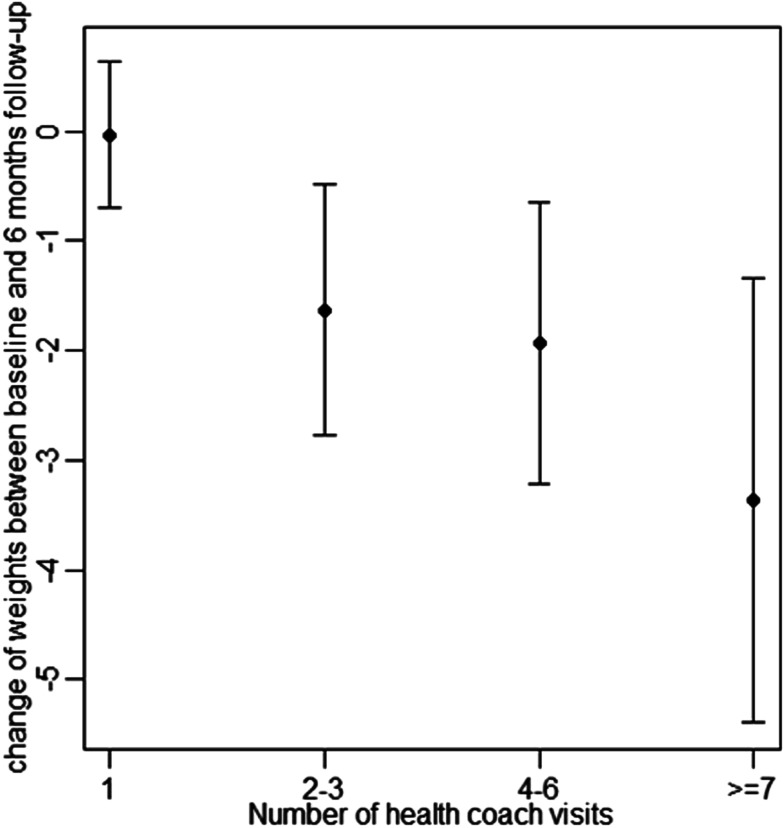
Variability of weight loss over six months compared to number of health coach visits.

In subgroup analyses examining associations of patient intervention exposure with weight change after 6 months, the number of completed health coaching visits was associated with statistically significant weight loss; the more coaching visits patients completed, the greater the weight loss (Table 3; Figure 1). Weight loss was not significantly associated with demographics or baseline clinical risk factors of prediabetes, diabetes, or hypertension. In the linear mixed-effects regression analysis evaluating predictors of weight reductions across the 3 time points (see Figure 1), a greater number of completed health coach visits predicted a greater reduction in weight after 6 months (*b* = −0.19, *SE* = .08, *P* = .013) (Figure 2).

**Table 3.** Association Between Patient Characteristics, Intervention Variables, and Changes in Weight (kg) From Baseline to 6 Months.

Measure	Mean Weight Value		Change	*P* Value^a^
	Baseline	6 Months		
Baseline BMI	​	​	​	0.011
Normal (n = 32)	62.44 (7.52)	62.87 (8.94)	0.43 (3.42)	​
Overweight (n = 56)	77.73 (9.67)	77.63 (10.82)	−0.10 (3.46)	​
Obese (n = 238)	108.50 (21.71)	106.89 (22.24)	−1.61 (5.83)	​
Prediabetes/Diabetes^1^	​	​	​	0.290
Yes (n = 162)	98.62 (23.64)	96.83 (23.53)	−1.79 (5.12)	​
No (n = 88)	99.25 (23.50)	98.16 (23.74)	−1.09 (4.92)	​
Prediabetes	​	​	​	0.360
Yes (n = 102)	97.09 (24.02)	95.35 (23.71)	−1.75 (4.94)	​
No (n = 88)	99.25 (23.50)	98.16 (23.74)	−1.09 (4.92)	​
Diabetes	​	​	​	0.218
Yes (n = 60)	101.22 (22.94)	99.35 (22.70)	−1.87 (5.46)	​
No (n = 304)	96.84 (25.31)	95.92 (25.25)	−0.91 (5.23)	​
Hypertension	​	​	​	0.982
Yes (n = 56)	108.70 (29.06)	107.62 (28.69)	−1.08 (4.18)	​
No (n = 308)	95.53 (23.63)	94.46 (23.58)	−1.07 (5.46)	​
Number of health coach visits	​	​	​	<0.001
1 (n = 171)	91.45 (23.45)	91.41 (23.91)	−0.04 (4.46)	​
2-3 (n = 113)	103.72 (26.98)	102.08 (26.54)	−1.63 (6.20)	​
4-6 (n = 49)	103.41 (23.52)	101.48 (23.85)	−1.93 (4.59)	​
7+ (n = 31)	99.59 (20.05)	96.23 (19.70)	−3.36 (5.74)	​

^a^*t* tests were used to examine associations with dichotomous variables, and analyses of variance (ANOVAs) were used to examine associations with categorical variables.

## Discussion

In this observational cohort analysis study, patients receiving at least 1 session of a registered dietitian-delivered health coaching visit through primary care had weight loss (>.5 kg) and improvements in other health outcomes after 3- and 6- months of follow-up. The number of completed coaching encounters was also associated with weight loss. To our knowledge, this is the first evaluation of a health coaching intervention delivered by registered dietitians and embedded within primary care.

Patients showed significant weight loss 3 months after their initial health coaching encounter and sustained after 6 months. There were also significant mean reductions in BMI after both follow-up time points. This finding underscores the extant literature on the positive impacts of health coaching on biophysical outcomes of individuals with chronic health conditions, and on weight management in particular (c.f., systematic review on short-term effectiveness of health coaching across various physiological, psychological, and behavior outcomes^18^). One RCT examining health coaching as compared to usual care among a higher-risk sample of patients with poorly controlled diabetes, hypertension, or hyperlipidemia demonstrated that clinical outcomes of weight showed persistent improvements after 1 year.^19^ Importantly, a greater number of completed coaching encounters in the present study was associated with greater weight loss, which further underscores the coaches’ positive impact, though also points towards the need for strategies to support retention. Regarding the number of health coaching visits and weight reduction, we saw the greatest difference in weight loss when patients had at least 2 visits (1.6 kg), compared to 1 (0.04 kg). We also saw a large difference between those with 4 to 6 visits (1.9 kg) and those with 7 or more (3.4 kg). Although our results indicate a positive relationship between increased health coaching visits and greater weight reduction, a functional follow-up would be to test the number and timing of coaching sessions while assessing both health and retention outcomes.

Among patients with diabetes or prediabetes, reductions in HbA1c values exceeded the minimum clinically significant difference after 6 months (i.e., reductions of 0.55% over 6 months). This estimate was largely driven by patients with a diagnosis of Type 2 diabetes; patients with prediabetes did not experience as strong of an effect. We hypothesize that those with diabetes may have had increased motivation to address health behaviors that specifically impact blood sugar; however, the number of participants with prediabetes was small and should be interpreted with caution. Additionally, the literature suggests that those with a higher A1c are more likely to see a larger reduction in blood sugar in response to behavioral intervention independent of a regression to the mean phenomenon.^20,21^ Another longitudinal study of individualized wellness coaching in adult primary care patients with prediabetes found significant improvements in health behaviors (e.g., aerobic exercise, healthy eating) after 3 and 6 months.^22^

Changes in blood pressure among patients who presented with hypertension, although trending in the right direction, were non-significant. This is likely due to the small sample and power limitations. It is also possible that 3 and 6 months of follow-up are too early for meaningful changes to occur; other work has shown significant impacts of health coaching among adult primary care over longer follow-up periods of 24 months. Additionally, because most patients reported weight loss as their primary goal, these patients’ coaching sessions may not have been targeted at strategies to improving blood pressure (e.g., sleep or stress reduction) and consequently did not impact this outcome. There is also the factor that weight loss was smaller than is typically necessary (>5%) to positively impact blood pressure. This subgroup of participants was small, however, and comparisons should therefore be interpreted with caution.

## Limitations

We tested the utility of health coaching integrated within primary care which provides pragmatic and generalizable information for other health care organizations but lacked a comparison group to determine the relative effects of health coaching on patient outcomes. In addition, we examined dose and health outcome relationships rather than examining the number and timing of sessions defined a priori which limits the confidence in our results.

Although our findings support the effectiveness of primary care-embedded health coaching and more visits yielded more weight loss, the optimal dose and timing of coaching encounters is unknown. Future evaluations should include identifying the optimal dosing and the best delivery mode (e.g., in-person vs telehealth), and which patients benefit from which approach. Randomized trials with more diverse patient populations are needed to evaluate effectiveness.

## Conclusion

Although primary care plays a critical role in chronic disease prevention through screening and education, patients’ lifestyle behaviors outside the formal clinical setting are strong determinants of health that require a higher-touch approach to address than is feasible in this setting. Health coaching is a promising approach, but models utilizing dietitian-trained health coaching embedded within this setting have not been evaluated. Our evaluation of a primary care-based health coaching intervention suggests that adding registered dietitians as extended care team members has the potential to improve patients’ weight and other cardiometabolic outcomes, and that these improvements can be sustained over 6 months.
